# Upgrading Nursing Students’ Foreign Language and Communication Skills: A Qualitative Inquiry of the Afterschool Enhancement Programmes

**DOI:** 10.3390/ijerph18105112

**Published:** 2021-05-12

**Authors:** Luis Miguel Dos Santos

**Affiliations:** Department of Global Convergence Management, Finance and Tax Management, Endicott College, Woosong University, Daejeon 34514, Korea; luismigueldossantos@yahoo.com; Tel.: +82-010-3066-7818

**Keywords:** British university student, Chinese, Chinese as a foreign language, learning Chinese, nursing student, social cognitive career theory, United Kingdom

## Abstract

Learning a foreign language is not easy for many students, particularly for nursing students who need to complete their internships in the hospital. However, motivation always drives them to the foreign language classrooms. The purpose of this study was to understand the motivations and reasons behind why these nursing students decided to study Mandarin Chinese as part of their personal development and enhancement beyond the curriculum. One main question and one sub-research question were written, why would nursing students decide to take Chinese as the tool for foreign language and culture development? What and why are the reasons and motivations for nursing students to take Chinese beyond their curriculum? The qualitative case study method was employed in order to investigate 36 nursing university students in the United Kingdom. The results of this study concluded that nursing students tend to study Chinese due to personal development and career goals. School leaders, department heads, government leaders, policymakers, human resources professionals, vocational trainers, and researchers may take this study as the opportunity to reform their current human resource and education plans to offer foreign language courses to university students, members of the public, learners, and interested parties for both personal development and career enhancements.

## 1. Introduction

In recent decades, due to ideas of globalisation and the economic growth of Chinese business communities and markets, the Chinese language has become a popular foreign language in many schools, universities, and graduate schools internationally. According to a recent report [1], Spanish (21%), French (10%), Italian (7%), German (5%), and Japanese (3%) were ranked as the top five languages for British students to learn in the United Kingdom. More than half of the participants believed that it is important for British students to understand at least one foreign language beyond the basic conversation level, as this may increase their skills and abilities during the post-Brexit era. In 2018, one-fourth of the participants indicated that they might encourage their children to learn a foreign language at school, with Mandarin Chinese as one of the most popular targets in the United Kingdom. Besides personal growth, many previous studies indicated that Chinese markets and business environments had grown rapidly all across the globe. Therefore, the knowledge and skill in Mandarin Chinese language and culture may allow individuals to access international opportunities and promotions.

In addition to the personal enhancement of foreign language learning, the skills of Mandarin Chinese could be helpful in the United Kingdom. According to the Higher Education Statistics Agency [2], Chinese students have become the largest international student group in the United Kingdom. During the 2014/2015 academic year, 89,540 Chinese students were enrolled at schools in the United Kingdom. Based on these statistics, almost one-fifth of the international student population in the United Kingdom comprised Chinese students in 2014/2015. However, the Chinese student population has significantly increased within a few years. In the 2018/2019 academic year, 35% of the non-European Union students came from China, which represented 35% of the total international student population (120,385). Although Indian students were ranked as the second highest international student population, the overall number was significantly lower than that of Chinese students, with only 26,685 Indian students during the 2018/2019 academic year.

Internationally, according to a recent report [3], for both first language speakers and foreign language speakers, the most spoken languages worldwide are English (1268 million), followed by Mandarin Chinese (1120 million), Hindi (637 million), Spanish (538 million), French (277 million), Standard Arabic (274 million), Bengali (265 million), Russian (258 million), Portuguese (252 million), Indonesian (199 million), Urdu (171 million), Standard German (132 million), Japanese (126 million), Swahili (99 million), and Marathi (95 million). Mandarin Chinese and related languages, such as its dialects, are not merely spoken by Chinese residents but also by people in other countries and regions. In fact, residents in mainland China, Hong Kong, Macau, Malaysia, Taiwan, and Singapore generally consider the Chinese language as one of the most spoken languages in their communities. Furthermore, significant groups of the Chinese diaspora and overseas residents living in communities in different parts of the globe may use the Chinese language for daily activities. Therefore, Mandarin Chinese and related languages are significantly useful in many backgrounds and situations.

Learning a foreign language is an important developmental step for public health professionals, particularly frontline nursing professionals who encounter patients [4]. A previous study [5] indicated that English as a second language (ESL) nursing students may face cultural conflict and confusion due to both language and cultural differences. Therefore, many of them would like to learn the English language in order to provide better care and services to their patients. The current clinical environment means that services are offered not only to local patients but also to foreign patients from different countries and regions [6]. Beyond healthcare and medical skills, one of the ways for medical staff to upgrade their soft skills is to gain knowledge of a foreign language. Effective communication regarding the patients’ health and medical issues always enhances the satisfaction with and results of medical treatment [7]. Although translators and interpreters may be available in some large hospitals, patients’ rights and treatments may suffer from medical staff not knowing how to explain the steps and procedures to them. More importantly, patients in rural communities who cannot access interpreters may not be able to receive appropriate information due to the lack of language and cultural awareness of the medical staff [8].

From the perspective of cultural awareness, some previous studies indicated that nursing professionals learn foreign languages as one of their professional skills, as they would like to understand the responses and needs of their patients [9]. A previous study [10] investigated a group of 401 clinical nurses from 21 hospitals regarding their understanding of and motivation for foreign language and culture learning. The results indicated that nurses’ understanding of foreign cultures and cultural awareness are key to responding to the needs of their patients. More importantly, due to globalisation and the mass movement of people [11], current clinical environments should be upgraded to diverse and inclusive sites in order to satisfy the needs of patients from different parts of the world [12].

The current healthcare environment is facing the highly demanding situation of intercultural and international interactions between local health professionals and foreign patients. A previous study [13] assessed and compared clinical nurses’ understanding and awareness of cultural and international patients and arrangements (i.e., from the perspectives of their knowledge and acceptance of first language and second language). The result indicated that nurses without upper-level second language skills and proficiencies might feel less confident when working with patients due to their lack of cultural awareness.

### 1.1. Purpose of the Study

Although many previous studies [14] indicated that understanding a foreign language and gaining cultural awareness from patients are key to increasing the outcomes of treatments, many nursing schools in the United Kingdom do not implement any foreign language requirements and elements into their current plans. Current curriculum plans are responding neither to the recommendations from numbers of reports nor to the needs of patients [9].

Although department heads and school leaders cannot change the curriculum plans for nursing students due to governmental policies, some schools decide to offer additional foreign language modules and training in afterschool programmes. Based on the current diverse population and demands of Chinese speaking professionals in the United Kingdom, a large number of schools and universities are offering Mandarin Chinese language courses for their students. According to The British Association for Chinese Studies [15], there are more than 30 British universities offering Chinese language and culture courses at the degree level in the United Kingdom. Moreover, most universities provide non-degree level language courses and modules at their language centres and departments. Therefore, university students may enjoy foreign language learning and training in most school environments in the United Kingdom.

The purpose of this study was to understand the motivations and reasons behind why these nursing students decided to study Mandarin Chinese as part of their personal development and enhancement beyond the curriculum. There are two significant points for this study. First, unlike Spanish and French (i.e., two of the most popular foreign languages in the United Kingdom), Mandarin Chinese does not share significant similarities with the English language. Therefore, the researcher wanted to understand why Mandarin Chinese would be their choice. Second, nursing students’ curriculum and plans are busy and packed due to heavy workload and multiple internships and practicums. The researcher wanted to understand why nursing students would decide to take additional foreign language courses and training, in this case, Mandarin Chinese. The research was guided by two research questions (i.e., one main research question and one sub-research question), which are:

1. Why would nursing students decide to take Mandarin Chinese (i.e., rather than other foreign languages) as the tool for foreign language and culture development?
  1.1 What are the reasons and motivations for nursing students to take Mandarin Chinese courses and training beyond their curriculum and plans, and why?

### 1.2. Theoretical Framework

Motivation is one of the most important elements in foreign language learning [16]. According to Bandura [17], individuals need to have reasons for motivation and direction in order to gain new knowledge and learning, particularly for adults who need to learn new knowledge beyond the compulsory curriculum [18]. Therefore, understanding the differences between groups and students may help both teachers and students to locate the appropriate teaching and learning methods, directions, and even the foreign language itself.

In this study, the researcher employed the social cognitive career theory [19,20,21] as the theoretical framework for investigating the motivational reasons behind why nursing students decided to learn Chinese as a foreign language during their university voyage. The social cognitive career theory [19,20,21] advocated that individuals developed their career and occupation goals and directions based on the social-cultural background of their living communities. In fact, the social cognitive career theory [19,20,21] was originally developed for the occupational-oriented issues, such as the reasons and motivations why individuals decided to start or leave their career and job in a certain way or direction. However, the framework of social cognitive career theory may be employed to understand the reasons and motivations why individuals decide to study and learn different courses and skills in our global communities [22]. Therefore, the current study employed the social cognitive career theory as the tool to understand and explore the reasons, motivations, and learning behaviours of nursing students in the United Kingdom, in this case, the Mandarin Chinese language [23].

**Figure 1:**
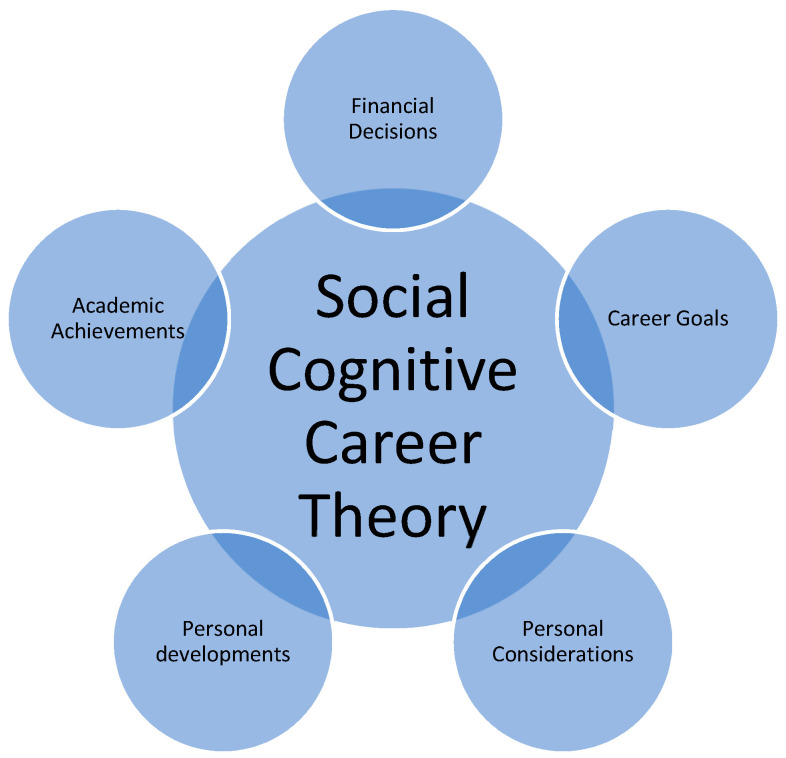
The social cognitive career theory.

Based on many studies and results [24,25,26], researchers have concluded that both intentions (e.g., individuals’ personal beliefs, personal development, ideas, and goals) [27,28] and behaviours (e.g., movements, conducts, and practices) [29,30] may influence an individuals’ reasons and motivations. Due to the social developments and changes, the current study employed the upgraded version of the social cognitive career theory [19,20,21,31]. The current version of the social cognitive career theory [31] was tested for a group of nursing students in the bilingual teaching and learning environment. With a similar background, the researcher advocated that it was appropriate for this study, particularly for the similar rationale. To conclude, five elements may influence the overall behaviours of the individuals, which are: financial decisions, career goals, personal developments, personal considerations, and academic achievements. To view the social cognitive career theory, please refer to Figure 1.

## 2. Materials and Methods

The researcher employed the qualitative research method to collect and analyse the data into themes and subthemes [32]. The current study collected data from a small group of individuals who were working on or completing their Mandarin Chinese courses as additional training beyond their curriculum (i.e., also known as the afterschool programme). As the nature of this study tended to collect data from a group of participants within a certain location and school, the researcher decided to employ the case study methodology.

### 2.1. Case Study

A qualitative method with the direction of a single case study research design was employed [33]. The case study approach was used because the design allowed for an in-depth understanding and exploration of the current topic about the foreign language learning in a designated site (i.e., school) and a study programme (i.e., nursing degree course). The performance of the case study allowed the researcher to understand the procedure, development, and responses of a group of participants in a particular area. Unlike phenomenological analyses, which may concern the behaviours and ideas of a large group of people in the cities, regions, and countries, a case study tends to focus on a particular site or group for in-depth understanding and investigation. In this case, since the researcher sought the reasons and motivations of a group of individuals in a particular site, this study fit the nature of the case study design.

### 2.2. The Afterschool Foreign Langauge Programme

Based on the current approval curriculum plan from the university and department of nursing, nursing students do not need to complete any foreign language and culture courses and training. However, the department leaders believed that nursing professional and medical staff should have a sense of cultural awareness of foreign and international patients’ behaviours. Therefore, the nursing school coordinated with the foreign language department and the language centre for the afterschool foreign language programme. Currently, besides Mandarin Chinese, the language centre offers Spanish, French, Italian, and German for students with a medical background (e.g., nursing students). As most of these learners are degree seekers in a particular subject, in this case, nursing students, the materials and knowledge of these courses are designed for specific purposes.

### 2.3. Participants and Recruitments

A total of 36 nursing students who are currently enrolled at one of the nursing training programmes (e.g., Bachelor of Nursing Studies) were invited to this study. These participants belong to a university in the United Kingdom. They are working on their Mandarin Chinese courses and training as an afterschool programme beyond their curriculum plan in the field of nursing. These participants are either second-year or final-year students with at least one year of Mandarin Chinese knowledge and training based on the afterschool programme.

### 2.4. Data Collection

The qualitative design was employed for the data collection procedure [34,35]. To increase the validity of the qualitative data, the researcher employed three sets of data collection tools, including interview sessions, focus group activities, and member checking interview sessions. Based on three different directions of the data sources, the researcher confirmed the data and responses [36].

First, the researcher started with the individual interview sessions for each participant. As most of the participants had packed schedules, the researcher offered either face-to-face interview sessions or distance-based interview sessions. All 36 participants selected the distance-based interview sessions. During the interview sessions, the researcher asked questions about their reasons and motivation for taking Mandarin Chinese as their foreign language. Each interview session lasted 48–62 min.

Second, after the researcher completed the individual interview sessions, the focus group activities started. As most of the participants agreed with the distance-based environment, the focus group activities were hosted online. Six participants were merged into one focus group activity. Therefore, six focus group activities were hosted based on student enrolment. Each focus group activity lasted 56–78 min.

Third, in order to confirm the validity of the data, the researcher sent the related data and responses to each participant for the distance-based member checking of the interview sessions. All participants read and agreed with their own responses. As a result, the research continued with agreement from the participants.

During the interview sessions and focus group activities, the researcher set up the digital recorder for data collection. All agreed that their voices and responses could be recorded for research purposes. However, visual data and videos were not captured.

During the data collection procedure, participants were allowed to use English and Mandarin Chinese for their responses and comments. As the researcher speaks both English and Mandarin Chinese, it would have been possible to translate and transcribe the voice messages from Mandarin Chinese to English. However, all participants shared their comments and opinions in English only. No translations services were needed.

### 2.5. Data Analysis

After the researcher collected all the essential information from the participants and different sessions, the researcher transcribed the voiced information to written transcripts. More than 300 pages of the written transcripts were created. The researcher read the data multiple times for categories and groups. First, based on the recommendation of different qualitative researchers and experts, the researcher decided to employ the open-coding technique [37,38]. The open coding technique allowed the researcher to categorise large size data and responses into systematic themes. As a result, after the employment of the open coding technique, the researcher merged 15 themes and 23 subthemes.

Qualitative researchers advocated that studies with a systematic direction should not have more than 10 themes and 10 subthemes. Therefore, after the completion of the open coding technique, the researcher employed the axial coding technique for further procedures [37,38]. Therefore, after several rounds of the techniques and studies, two themes and two subthemes were categorised.

### 2.6. Human Subject Protection

The personal and site protection is the most important element of this study. The researcher employed different ways to protect the privacy of all parties. Therefore, the signed agreements, voiced messages, written transcripts, contact information, email addresses, computer, data materials, and related materials were locked in a password-protected cabinet. Only the researcher had the rights to read the materials. After the researcher completed the study, the related materials were deleted and destroyed in order to protect the privacy of all parties. The site administrators indicated that the school information should be masked. Therefore, no detailed information of the site is disclosed. The study was conducted according to the guidelines of the Declaration of Helsinki and approved by The Section of Woosong University Academic Research Funding.

## 3. Results and Discussions

Although all students are studying the same bachelor’s degree programme at the same university, their family background, understanding, education, life experience, and expectations would be different. However, the reasons and motivations of learning Chinese as a foreign language bring all of them together into a learning cohort. Through the lens of social cognitive career theory, the researcher yielded two themes and two subthemes based on the responses of the participants. There were two research questions (i.e., one main research question and one sub-research question). Please refer to Table 1 for the themes and subthemes.

### 3.1. Mandarin Chinese as One of the Strongest Languages for Global Development

According to recent research [39] about the motivation for learning Chinese as a foreign language in the European Union, Chinese has become one of the popular foreign languages alongside English, Italian, and French. A previous study [40] also indicated that Chinese organisations and businesses are investing a lot in the European Union, increasing the demand for Chinese language and cultural awareness due to the increasing Chinese-speaking population. Based on the financial and economic development and influence of Chinese organisations in Europe, many participants decided to enrol in Chinese language courses and training in order to serve potential patients in the region. Two participants said,

*… there are many Chinese organisations and investments in Europe … it is a global trend to understand Chinese language and the related cultural behaviours … I would like to learn Chinese because I may enter the global business development in the future …*

(Participant #5, individual interview)

*… there are many Chinese investments in the United Kingdom and Europe … if I can speak Chinese, I may join the developing workforce in the East Asian region … the economy in the United Kingdom is very negative … if I can speak Chinese, I may have better opportunities …*

(Participant #28, focus group)

#### 3.1.1. Personal Development and Potential International Promotion

Based on the lens of the social cognitive career theory [19,20,21,31], besides regional development opportunities, many participants explained that seeking language proficiency and cultural awareness of Chinese language and culture may upgrade their soft skills and personal development. First, almost all indicated that they would like to learn a unique language and culture that is not the same as what others learn. For example, many stated that Spanish and French language proficiencies do not provide them with uniqueness among others. Several participants explained,

*… there are too many people who can speak Spanish and French on the street … but there are many Chinese and East Asian patients who beg for help in Mandarin Chinese … I think if I can speak the language … it may be a great opportunity for personal development …*

(Participant #11, individual interview)

*… I want to learn Chinese because I want to have a unique development … We have a lot of Chinese friends and residents in the United Kingdom … but most of the Mandarin Chinese speakers are Chinese or East Asian residents … I want to speak the Mandarin Chinese language as a non-East Asian … this experience is unique and will be beneficial to my personal enhancement …*

(Participant #13, individual interview)

*… Chinese culture is not only fun … but also meaningful and excited … each Chinese character represents a historical story … unlike the current English words and sentences … Chinese is unique … I want to learn and enjoy this history in the Asian region …*

(Participant #16, individual interview)

With the reflection of a previous study [41], the current finding indicated that in addition to the university education and vocational skills, traditional-age students have the curiosity to explore different opportunities and chances for personal development. Based on the lens of the social cognitive career theory [19,20,21,31], the element of personal development may be positioned in this direction.

Second, a great number of participants indicated that Mandarin Chinese might be connected to some alternative treatments [42]. Several indicated that for pain symptoms, some medical professionals are recommending the cupping treatment based on traditional Chinese medicine. Such oriental treatments, therefore, increased their interests in understanding Chinese language and culture. One participant remarked,

*Some alternative and oriental treatments are very useful for pain management … as I would like to learn more ideas from these alternative treatments, I learn to learn the roots of these … as many of the treatments are from the traditional Chinese medicine … I want to gain the language and cultural proficiency …*

(Participant #36, focus group)

Third, more than half of the participants indicated that they want to travel or work overseas after their graduation from university. Based on the lens of the social cognitive career theory [19,20,21,31] and the element of career goals, in order to increase their chances of international promotions and opportunities, many of these scholarships and grants may require foreign language proficiency. Several participants said,

*There are many scholarships, and international development programmes are hosted by the Chinese agencies … I want to gain the scholarships and work in China for international development … so I want to learn Chinese … to prepare myself …*

(Participant #8, focus group)

*In the future, I want to do some international charity works … there are many opportunities in the Chinese speaking regions, such as mainland China or some rural communities in Malaysia … they want to have some Chinese speaking volunteers … I want to be prepared for this international opportunities …*

(Participant #19, focus group)

In short, almost all participants believed that Chinese language proficiency and cultural awareness would bring them various types of personal development and career opportunities. Based on the lens of the social cognitive career theory [19,20,21,31], the results of these themes and subthemes indicated that young nursing professionals would like to expand their horizons with various scholarships and opportunities to other countries and regions. The elements of personal development and career goals therefore nominated the reasons and motivations of learning Chinese as a foreign language among this group of nursing students. Unlike individuals who completed their university education and training several decades ago, youths tend to use their vocational skills and language proficiencies as tools to expand their horizons to different opportunities [43].

### 3.2. The Multitude of Mandarin Chinese-Speaking Patients

According to the Higher Education Statistics Agency [2], the international student population in the United Kingdom has been increasing rapidly. Currently, nearly 25% of the international student population is Chinese. Although there are no contemporary statistics (i.e., the current governmental census was established almost a decade ago) regarding how many Chinese residents are living in the United Kingdom, the government indicated that there were 393,141 ethnically Chinese people living in England and Wales in 2011 [44]. However, based on the growing population from the Higher Education Statistics Agency [2], it is expected that Mandarin Chinese speakers have increased over the past decade. Reflecting the results of a previous study, nurses and medical staff tend to seek understanding of additional foreign languages and cultures in order to provide excellent services to their patients. Several participants explained,

*Besides vocational and medical skill training, I think the medical staff in the United Kingdom should be trained as bilingual and bicultural professionals in order to meet the needs of our patients …*

(Participant #3, individual interview)

*… If I can speak Chinese … I can serve not only Chinese patients but also many patients from the East Asian region as many speak Chinese … I can help a greater number of people …*

(Participant #31, focus group)

*… Chinese residents are one of the important groups of people in our community … if I can speak the language, I can provide additional help and care to more people …*

(Participant #23, individual interview)

#### 3.2.1. Cultural Awareness of the Mandarin Chinese Language: Cultural Understanding and Practices beyond Chinese People

Many previous studies [45] indicated that cultural awareness and intercultural understanding serve as some of the keys to patient care, as they allow medical staff to understand why and how patients behave in certain ways [46]. Many schools in the United Kingdom offer cultural sensitivity training programmes in order to increase the open-mindedness and cultural awareness of both pre-service and in-service medical staff for multicultural interaction.

From the perspective of the social cognitive career theory [19,20,21,31] and the element of career goals, based on the findings of this study, students are taking foreign language courses with cultural awareness training (i.e., in this case, Mandarin Chinese) in order to increase their understanding of multiculturalism [47]. Almost all participants expressed that the language courses in Mandarin Chinese increased their oral and communication skills and proficiencies in interacting with patients with East Asian backgrounds. Two participants described,

*… just the word painful … there are several different levels and explanation … before I joined the nursing school, I could not explain the level in detail … so the Chinese patients … I can serve as the tool to interpret the symptoms of my patients …*

(Participant #34, focus group)

*… cultural exchanging and interaction are important in patient communication … many hospitals need to hire interpreters … but if we can speak the language, the overall process and duration will be reduced …*

(Participant #22, individual interview)

Besides ideas about communication, many indicated that cultural differences regarding care are important. Based on the current findings and those of a previous study [45], the participants have the motivation to learn language, cultural understanding, and communication skills from their foreign language courses (i.e., in Mandarin Chinese) in order to gain cultural competencies.

Moreover, under the themes and subthemes of this study, the researcher concluded that personal development and career goals highly influence the participants’ reasons and motivation of learning Chinese as a foreign language. More importantly, all participants expressed that they are willing to learn additional cultural competencies in order to offer additional services to their patients. Many have changed their minds about cultural awareness, groups’ behaviours, and personal decisions after the completion of their foreign language training.

## 4. Limitations and Future Research Directions

Each study should have its own limitations. However, each limitation allows for further future developments. The researcher identified three limitations. First, one of the directions for the current study was concerned about the reasons and motivations of nursing students. However, other students, programmes, and departments may have a similar problem for their students and programmes. Therefore, future researchers may take this study as an opportunity for their own departments and programmes.

Second, the current study has a focus on a particular university’s nursing degree programme. However, a further study with a larger focus, such as regional studies and national studies, may be conducted. Therefore, future researchers may develop a study with the national data collection procedure.

Third, there are multiple foreign language courses and training programmes at the university level. In this case, the researcher focused on training in the field of Mandarin Chinese language and culture knowledge. Future researchers may take this study as the opportunity to study other foreign languages, particularly minority languages in the United Kingdom.

## 5. Conclusions

Learning a foreign language is not an easy step for many individuals and students, particularly for nursing students who need to study and complete their internships in the hospital. However, motivation always drives them to the foreign language classrooms. In this study, based on the lens of social cognitive career theory [19,20,21,31], the results of this study concluded that nursing students tend to study their Mandarin Chinese courses and training due to the reasons and motivations of personal development and career goals.

Throughout the study, the researcher concluded that participants sought to learn Mandarin Chinese due to the global development, personal development, and international promotion and for Mandarin Chinese-speaking patients, the cultural understanding, and practices of the Chinese people. It is worth noting that personal development and career goals nominated the reasons and motivations of learning Mandarin Chinese as a foreign language among this group of nursing students. The responses and comments were intensive and rich, which met the directions and ideas of the theoretical framework.

Based on the responses and comments from the users of the programme (i.e., nursing students in this case), school leaders, department heads, government leaders, policymakers, human resources professionals, vocational trainers, and researchers may take this study as the opportunity to polish and reform their current human resources and education plans in order to offer foreign language courses and training to university students, members of the public, learners, and interested parties for both personal development and career enhancements. Without a doubt, foreign language skills are one of the most important factors for pre-service and in-service nursing professionals. If the universities provide effective training and courses for our medical professionals during their university voyage, our medical staff’s communicational and interactive skills will be increased effectively. Patients without established foreign language skills will be beneficial to the development of these types of foreign language programmes for medical professionals.

## Figures and Tables

**Table 1:** Themes and subthemes of the study.

Themes and Subthemes of This Study		
Section 3.1.		Mandarin Chinese as one of the Strongest Languages for Global Development
	Section 3.1.1.	Personal Development and Potential International Promotion
Section 3.2.		The Multitude of Mandarin Chinese-Speaking Patients
	Section 3.2.1.	Cultural Awareness of the Mandarin Chinese Language: Cultural Understanding and Practices Beyond Chinese People

## References

[1] Kennedy K. Spanish Tops Languages to Learn in 2018, Says British Council. https://thepienews.com/news/learning-new-language-popular-resolution-2018-says-british-council-survey.

[2] Mantle R. (2020). Higher Education Student Statistics: UK, 2018/19.

[3] Duffin E. (2020). The Most Spoken Languages Worldwide in 2019.

[4] Dos Santos L.M. (2019). Evaluation checklist for English language teaching and learning for health science professionals. World Trans. Eng. Technol. Educ..

[5] Salamonson Y., Everett B., Koch J., Andrew S., Davidson P. (2008). English-language acculturation predicts academic performance in nursing students who speak English as a Second Language. Res. Nurs. Health.

[6] Schouten B.C., Meeuwesen L. (2006). Cultural differences in medical communication: A review of the literature. Patient Educ. Couns..

[7] Hewett D.G., Watson B.M., Gallois C., Ward M., Leggett B.A. (2009). Communication in medical records: Intergroup language and patient care. J. Lang. Soc. Psychol..

[8] Meuter R.F.I., Gallois C., Segalowitz N.S., Ryder A.G., Hocking J. (2015). Overcoming language barriers in healthcare: A protocol for investigating safe and effective communication when patients or clinicians use a second language. BMC Health Serv. Res..

[9] Bardet A., Green A.R., Paroz S., Singy P., Vaucher P., Bodenmann P. (2012). Medical residents’ feedback on needs and acquired skills following a short course on cross-cultural competence. Int. J. Med. Educ..

[10] Chae D., Park Y., Kang K., Kim J. (2020). A multilevel investigation of cultural competence among South Korean clinical nurses. Scand. J. Caring Sci..

[11] Crisol Moya E., Caurcel Cara M.J. (2020). Percepciones de los estudiantes de la especialidad de lengua extranjera: Inglés sobre atención a la diversidad en la formación inicial del profesorado de Educación Secundaria. Onomázein Rev. lingüística Filol. y traducción.

[12] Stilwell B., Diallo K., Zurn P., Vujicic M. (2004). Migration of health-care workers from developing countries. Bull. World Health Organ..

[13] Gasiorek J., van de Poel K. (2018). Language-specific skills in intercultural healthcare communication: Comparing perceived preparedness and skills in nurses’ first and second languages. Nurse Educ. Today.

[14] Dos Santos L.M. (2020). The challenges of public health, social work, and psychological counselling services in South Korea: The issues of limited support and resource. Int. J. Environ. Res. Public Health.

[15] Chinese in UK Universities. http://bacsuk.org.uk/chinese-in-uk-universities.

[16] You C., Dörnyei Z. (2016). Language learning motivation in China: Results of a large-scale stratified survey. Appl. Linguist..

[17] Bandura A., Cervone D. (1983). Self-evaluative and self-efficacy mechanisms governing the motivational effects of goal systems. J. Pers. Soc. Psychol..

[18] Dos Santos L.M. (2018). The cultural cognitive development of personal beliefs and classroom behaviours of adult language instructors: A qualitative inquiry. Brain Sci..

[19] Lent R.W., Brown S.D., Hackett G. (1994). Toward a unifying social cognitive theory of career and academic interest, choice, and performance. J. Vocat. Behav..

[20] Lent R.W., Brown S.D. (1996). Social cognitive approach to career development: An overview. Career Dev. Q..

[21] Dos Santos L.M., Islam N. (2018). Foreign language learning beyond English: The opportunities of One Belt, One Read (OBOR) Initiative. Silk Road to Belt Road.

[22] Dos Santos L.M. (2019). Recruitment and retention of international school teachers in remote archipelagic countries: The Fiji experience. Educ. Sci..

[23] Dos santos L.M. (2020). Becoming university language teachers in South Korea: The application of the interpretative phenomenological analysis and social cognitive career theory. J. Educ. e-Learning Res..

[24] Dos Santos L.M. (2020). I teach nursing as a male nursing educator: The East Asian perspective, context, and social cognitive career experiences. Int. J. Environ. Res. Public Health.

[25] Tayan B. (2017). Students and teachers’ perceptions into the viability of mobile technology implementation to support language learning for first year business students in a Middle Eastern university. Int. J. Educ. Lit. Stud..

[26] Pavlova L., Vtorushina Y. (2018). Developing students’ cognition culture for successful foreign language learning. SHS Web Conf..

[27] Dos Santos L.M. (2020). I want to become a registered nurse as a non-traditional, returning, evening, and adult student in a community college: A study of career-changing nursing students. Int. J. Environ. Res. Public Health.

[28] Brown S., Lent R. (2019). Social cognitive career theory at 25: Progress in studying the domain satisfaction and career self-management models. J. Career Assess..

[29] Dos Santos L.M. (2020). International school science teachers’ development and decisions under social cognitive career theory. Glob. J. Eng. Educ..

[30] Dickinson J., Abrams M.D., Tokar D.M. (2017). An examination of the applicability of social cognitive career theory for African American college students. J. Career Assess..

[31] Dos Santos L.M. (2021). Developing bilingualism in nursing students: Learning foreign languages beyond the nursing curriculum. Healthcare.

[32] Merriam S.B. (2009). Qualitative Research: A Guide to Design and Implementation.

[33] Yin R.K. (2012). Applications of Case Study Research.

[34] Creswell J. (2012). Qualitative Inquiry and Research Design: Choosing among Five Approaches.

[35] Tang K.H., Dos Santos L.M. (2017). A brief discussion and application of interpretative phenomenological analysis in the field of health science and public health. Int. J. Learn. Dev..

[36] Clandnin D., Connelly F. (2000). Narrative Inquiry: Experience and Story in Qualitative Research.

[37] Thomas D.R. (2006). A general inductive approach for analyzing qualitative evaluation data. Am. J. Eval..

[38] Strauss A., Corbin J.M. (1990). Basics of Qualitative Research: Grounded Theory Procedures and Techniques.

[39] Morbiato A., Arcodia G.F., Basciano B. (2020). Topic and subject in Chinese and in the languages of Europe: Comparative remarks and implications for Chinese as a second/foreign language teaching. Chin. Second Lang. Res..

[40] Pepermans A. (2018). China’s 16 + 1 and Belt and Road Initiative in central and eastern Europe: Economic and political influence at a cheap price. J. Contemp. Cent. East. Eur..

[41] Flores L.Y., O’Brien K.M. (2002). The career development of Mexican American adolescent women: A test of social cognitive career theory. J. Couns. Psychol..

[42] Dos Santos L.M. (2019). The application of Chinese preventive medical treatments and activities: A qualitative collection of front-line traditional Chinese medicine doctors and medical professionals. J. Eng. Appl. Sci..

[43] Dos Santos L., Lo H.F. (2018). The development of doctoral degree curriculum in England: Perspectives from professional doctoral degree graduates. Int. J. Educ. Policy Leadersh..

[44] Chinese Ethnic Group: Facts and Figures. https://www.ethnicity-facts-figures.service.gov.uk/summaries/chinese-ethnic-group.

[45] Majumdar B., Browne G., Roberts J., Carpio B. (2004). Effects of cultural sensitivity training on health care provider attitudes and patient outcomes. J. Nurs. Scholarsh..

[46] Hultsjö S., Bachrach-Lindström M., Safipour J., Hadziabdic E. (2019). Cultural awareness requires more than theoretical education: Nursing students’ experiences. Nurse Educ. Pract..

[47] Shepherd S.M. (2019). Cultural awareness workshops: Limitations and practical consequences. BMC Med. Educ..

